# Differential diagnosis and surgical management of cecal dilatation *vis-a-vis* cecal impaction in bovine

**DOI:** 10.14202/vetworld.2018.1244-1249

**Published:** 2018-09-11

**Authors:** Gurnoor Singh, Rahul Kumar Udehiya, Jitender Mohindroo, Ashwani Kumar, Tarunbir Singh, Pallavi Verma, Nameirakpam Umeshwori Devi, Arun Anand

**Affiliations:** Department of Veterinary Surgery and Radiology, Guru Angad Dev Veterinary and Animal Sciences University, Ludhiana - 141 004, Punjab, India

**Keywords:** buffalo, cattle, cecum, percussion, typhlotomy, ultrasonography

## Abstract

**Aim:**

The present study was undertaken to study the clinical and hemato-biochemical alterations, ultrasonography, and surgical treatment of bovine suffering from cecal dilatation and cecal impaction.

**Materials and Methods:**

The present study was conducted on 11 bovines (9 buffaloes and 2 cattle) suffering from cecal dilatation (n=6) and cecal impaction (n=5). The diagnosis of surgical affections of cecum was made on the basis of clinical examination, hematobiochemistry, ultrasonography, and exploratory laparotomy.

**Results:**

A marked decrease in serum total protein, albumin, chloride, potassium, and calcium levels while an increase in lactate concentrations was recorded. Peritoneal fluid examination revealed an increase in total protein concentration. Per rectal examination along with ultrasonography was used as a confirmatory diagnostic tool for cecal dilatation and cecal impaction. Ultrasonographic features of cecal dilatation and cecal impaction were recorded. Left flank laparorumenotomy was performed in six animals with dilated cecum along with colonic fecalith. Post-rumenotomy, these animals were treated with massage of cecum along with kneading of colonic fecalith. Right flank typhlotomy was done in the remaining five animals having impacted cecum for decompression of the dilated cecum. 9 of 11 animals survived which underwent surgery and remained healthy up to 3-month follow-up.

**Conclusion:**

Ultrasonography was reliable in the diagnosis of cecal dilatation and cecal impaction in bovine. Left flank exploration after laparorumenotomy is an ideal surgical technique for the management of cecal dilatation, while right flank typhlotomy is ideal for the management of cecal impaction in bovine.

## Introduction

Cecal dilatation is a common and economically important abdominal disorder that affects mainly dairy animals, but the pathogenesis of the diseases remains poorly understood. The cecum may become abnormally dilated with gas or distended with ingesta and associated with partial or complete cessation of the passage of intestinal contents which further leads to complete absence of defecation [1].

Per rectal examination has been used as an important diagnostic tool to palpate dilatation or displacement of the cecum [2]. Cecal dilatation may also, secondarily, be associated with foreign body syndrome [3]. Radiographic evaluation of the cecum in bovine is not possible due to the massive viscera and body girth. Several studies claim ultrasonographic diagnosis of cecal dilatation in cattle [3-5]. However, there is no report on ultrasonographic diagnosis of cecal impaction in bovine. Early diagnosis and surgical intervention are required for achieving a successful outcome. Conservative treatment is not rewarding in the bovine with cecal affection and surgery should not be delayed in these patients [5].

The present study was undertaken to study the clinical and hemato-biochemical alterations, ultrasonography, and surgical treatment of bovine suffering from cecal dilatation and cecal impaction.

## Materials and Methods

### Ethical approval

This clinical study was duly approved by the Institutional Animal Ethics Committee.

### Animals, history, and physical examination

The study was conducted on 11 bovines (9 buffaloes and 2 cattle) confirmed for cecal dilatation (n=6) and cecal impaction (n=5). Permission to conduct the clinical study was granted by the Institutional Animal Ethics Committee. The diagnosis was made on the basis of clinical examination, hematobiochemistry, ultrasonography, and surgical findings. A detailed history of feed and water intake, duration of illness and fecal output, pregnancy status, pain, tympany, and abdominal distension were recorded in all the animals. Rectal temperature (°F), heart rate (beats/min), respiration rate (breaths/min), status of mucous membrane, and rumen motility were recorded [6]. Per rectal findings were recorded in all the animals.

### Hematological and biochemical analyses

Hematobiochemistry included estimation of hemoglobin (g%), total leukocytic count (10^3^/μL), differential leukocytic count (%), packed-cell volume (%), total protein (g/dL), albumin (g/dL), sodium (mmol/dL), potassium (mmol/dL), chloride (mmol/dL), calcium (mg/dL), phosphorous (mg/dL), and lactate (mmol/L) using VITROS DT-II Chemistry System (Ortho-Clinical Diagnostics, Johnson and Johnson Company). Peritoneal fluid samples were collected pre- or intraoperatively and were examined for total protein (g/dL), albumin (g/dL), and total leukocytic count (10^3^/μL).

### Ultrasonographic examination

Ultrasonographic examination of the dilated cecum was done from the right flank region using Logiq 3 BT Expert Ultrasound Machine, GE Healthcare with 5 MHz convex transducer. Cecal dilatation was confirmed when a gas-filled cecum was palpated during per rectal examination and gas was seen in more than half of the cecum on ultrasonogram. Cecal impaction was confirmed when a small gas cap along with doughy cecal body could be palpated during per rectal examination, and only a gas cap involving the dorsal part of cecum could be seen on ultrasonogram.

### Surgical management

In six animals with cecal dilatation, left flank laparotomy followed by rumenotomy was performed by giving a left mid vertical flank incision on standing animal under proximal paravertebral local anesthesia using 2% lignocaine hydrochloride. Foreign bodies, if any present in the reticulum, were retrieved. Rumen was closed after flushing in a routine fashion using absorbable suture material (No. 2 chromic catgut) in a double layer using Lambert pattern followed by Cushing pattern. After closure of the rumen, the right side was palpated, and fecaliths were kneaded. The cecum was blindly palpated after passing the hand into the abdomen. The body of the cecum was massaged and kneaded with the help of fist for 2-3 min, and the left flank incision was closed in routine fashion. In five animals with cecal impaction, right flank typhlotomy was performed by giving a right mid ventral flank incision on standing animal under linear infiltration of local anesthesia using 2% lignocaine hydrochloride. After incising muscles and peritoneum, greater omentum was retracted cranially for the exteriorization of the impacted cecum. Proper packing with surgical drapes was done around the exposed cecum to prevent contamination of the abdominal cavity. A 4-5 cm-long incision was made on the apex of the cecum, and the impacted contents were retrieved. The cecal incision was closed using monofilament absorbable suture material (Polyglactin 910, 2-0) in a double layer using a simple continuous suture pattern followed by Cushing suture pattern. Follow-up was taken up to 3 months in all the animals.

### Statistical analysis

The quantitative data were presented as mean±standard error (SE) and qualitative data as frequency or percentage. The statistical study was estimated using independent t-test in SPSS version 16, (IBM. USA) for Windows.

## Results

**Figure-1:**
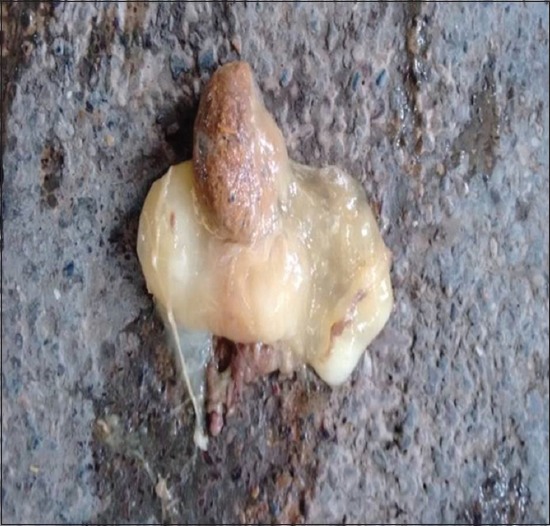
Photograph showing scanty mucoid feces.Discussion

**Figure-2:**
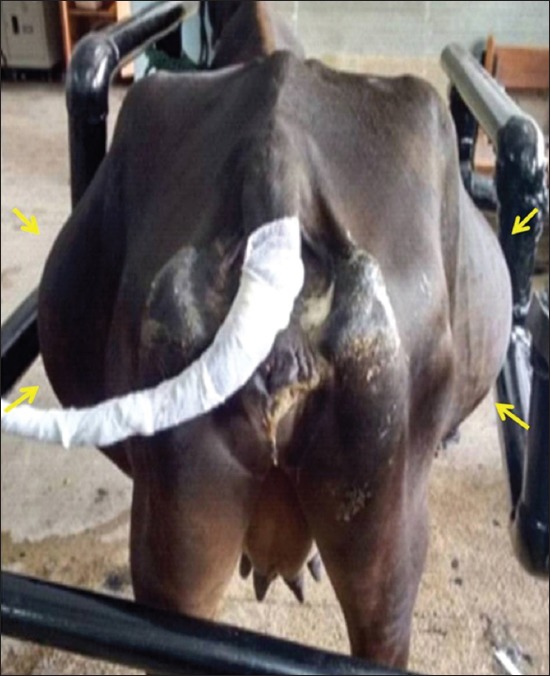
Photograph showing bilateral distension in cattle suffering from cecal dilatation.

**Figure-3:**
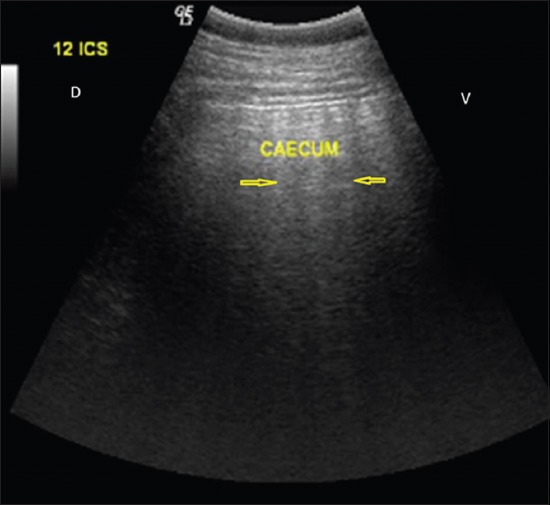
Dorsal (D) and ventral (V) aspect photograph showing thick crescent-shaped line cecum at 12th intercostal space with prominent reverberation artifacts suggestive of gas-filled cecum. D and V aspect.

**Figure-4:**
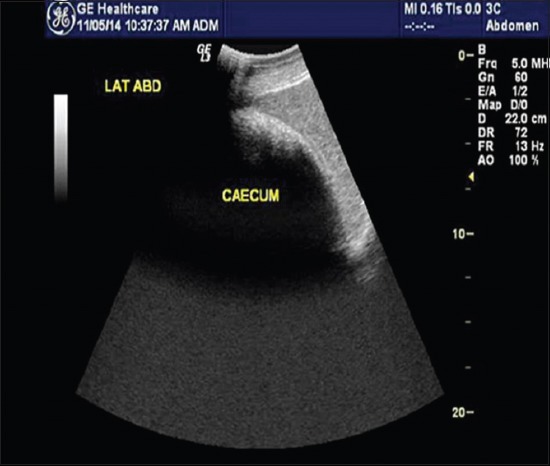
Ultrasonogram showing a distended semicircular structure with strong distal acoustic shadow on the lower right flank suggestive of cecal impaction.

**Figure-5:**
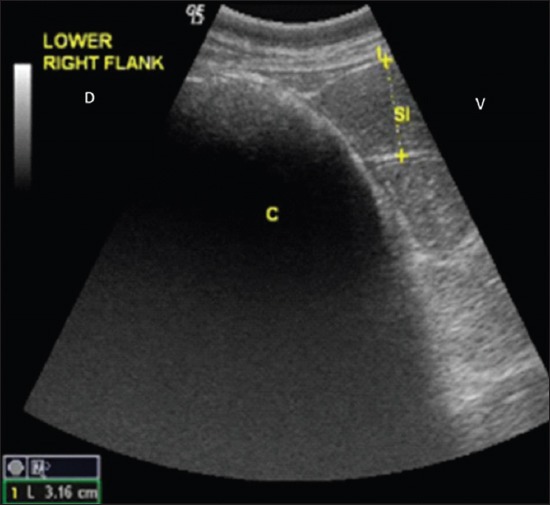
Ultrasonogram showing a dilated semicircular structure with strong distal acoustic shadow with distended intestinal loop is visible cranial to cecum on the lower right flank.

**Figure-6:**
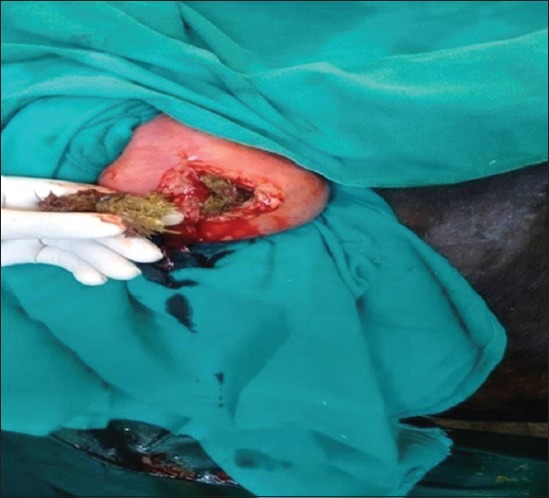
Photograph showing exteriorization of impacted cecum during right flank laparotomy.

Among the 11 bovines included in the present study, all were in the middle-age group and were female with a mean age of 5.72±0.53 years. Complete anorexia was recorded in majority of the animals (n=7, 63.63%), whereas reduced appetite was seen in the remaining four animals. Water intake was normal in majority of animals (n=9, 81.82%), whereas reduced water intake was seen in the remaining two animals. Pain was absent in most of the animals (n=7, 63.63%), while a single episode of pain was observed in the remaining four animals. Duration of illness ranged from 4 to 10 days with a mean duration of 6.18±0.53 days in all the animals. Complete loss of defecation with or without passing mucus (Figure-1) was observed in majority of the animals (n=9, 81.82%), whereas scanty/mucoid feces were seen in the remaining two animals. Of 11 animals, three were non-pregnant, four were in early pregnancy (pregnancy <6 months), and four were in advanced pregnancy (pregnancy more than 6 months). Tympany was absent in majority of animals (n=8, 72.73%), while in three animals (n=3, 27.27%), tympany was reported by the owner. Abdominal distension (Figure-2) was present in majority of animals (n=9, 81.82%) while absent in two remaining animals. Mucus membrane was normal in six cases, congested in two animals, and pale in three animals. Ruminal motility was reduced in all the animals. Ping sound was recorded at the caudodorsal half of the right paralumbar fossa on simultaneous auscultation and percussion in all the cases of cecal dilatation. However, in animals with cecal impaction, the ping sound could not be auscultated. During per rectal examination in animals suffering from cecal dilatation, a large distended cecum could be palpated on the right side which was prominently filled with gas, and the fingers bounced back on application of pressure. In animals suffering from cecal impaction, a large distended cecum could be palpated on the right side which appeared to be prominently filled with ingesta and was doughy in consistency. In either of the cecal affections, there was a complete absence of feces, the apex of the cecum was displaced toward or into the pelvic inlet, and dilated intestine loops were palpable in the right flank region. All the animals suffering from cecal dilatation and cecal impaction showed normal rectal temperature (100.58±0.50°F), heart rate (81.63±5.55 beats/min), and respiratory rate (24.09±1.32/min). Hematobiochemistry findings revealed normal hemoglobin (11.26±0.32 g%) and packed cell volume (31.60±1.1%) of all animals with cecal affection which were within the normal reference range. The total leukocytic count was in the high normal range (11.72±1.683×10^3^/μL) along with marked neutrophilia (71.54±1.71%) and lymphopenia (28.45±1.71%). Hypoproteinemia (5.89±0.24 g/dL), hypoalbuminemia (2.37±0.27 g/dL), hypokalemia (3.24±0.16 mmol/L), hypochloremia (90.81±4.9 mmol/L), and hypocalcemia (7.53±0.23 mg/dL) were recorded in all animals. Sodium and phosphorous levels were within the reference range for healthy animals (138.71±5.37 mmol/L and 3.24±0.16 mg/dL), while lactate was slightly increased in all the animals (2.73±0.23 mmol/L). Peritoneal fluid examination could be collected pre- or intraoperatively in five animals only. An increase in the mean±SE value of total protein (3.15±0.04 g/dL), a decreased albumin (1.26±0.06 g/dL), and normal total leukocytic count (48.348±4.804×10^3^/μL) were recorded in all cases. On ultrasonographic examination of six animals suffering from cecal dilatation, the cecum could be visualized from the dorsal right flank up to 11^th^ intercostal space. The ventral border of cecum could be visualized up to distal third of the rib cage in all the animals. The cecal wall could be seen as a thin echogenic line with line reverberations distal to the wall, making it impossible to visualize the structure/contents of the abdomen (Figure-3). In animals suffering from cecal impaction, a distended cecum could be visualized as a crescent-shaped structure having an extent similar to cecal dilatation along with strong distal acoustic shadowing (Figure-4). Dilated small intestine loop could be seen cranial and ventral to the impacted cecum (Figure-5). Left flank laparorumenotomy was performed in six animals suffering from cecal dilatation following evacuation of rumen along with retrieval of foreign bodies (n=2). Post-rumenotomy, the cecum was massaged with kneading of colonic fecalith in all cases. Of six animals, five showed good recovery while one died after a few weeks. Right flank typhlotomy was done in five animals diagnosed with cecal impaction based on per rectal and ultrasonographic findings. Cecum was decompressed, and flushing was done (Figure-6). The cecum was closed in routine manner as described in material and method. One animal collapsed during surgical intervention, while four animals recovered after typhlotomy. All the animals suffering from cecal dilatation and cecal impaction started passing feces after surgery. ↱All the nine animals which survived remained healthy up to 3-month follow-up.

## Discussion

Cecal dilatation is usually diagnosed on clinical findings and treated conservatively or surgically [5]. Earlier workers also reported cecal dilatation and cecal impaction in middle-aged animals [7]. Khalphallah *et al*. [1] stated that buffaloes aged 3-7 years had the highest frequency of cecal dilatation. Complete anorexia and reduced water intake in animals suffering from cecal affections may occur due to sudden changes in feeding, swallowing of indigestible foreign bodies, and changes in rumen microflora with prolonged and excess use of antibiotic [7]. The absence of pain in most of the animals with a single episode of pain in few cases corroborates to the findings of Fubini *et al*. [8]. The longer duration of illness could be due to longer attempts of self-medication or symptomatic treatment by local veterinarians. Complete loss of defecation with or without passing mucus in majority of the animals corroborated with the findings of Braun *et al*. [5]. Pregnancy did not seem to be related with cecal dilatation and cecal impaction [7]. Reduced rumen motility in all the animals with cecal affection has also been reported by Braun *et al*. [2] who suggested that, sometimes, rumen contractions may persist with decreased amplitude during the ↱disease. Ping sound was recorded at the caudodorsal half of the right paralumbar fossa on simultaneous auscultation and percussion in all the cases of cecal dilatation which corroborated with the findings of Fubini *et al*. [8], Braun *et al*. [2], and Braun *et al*. [5]. A positive ping sound on percussion auscultation at the caudodorsal half of the right paralumbar fossa was an important finding because it occurred in 100% of animals suffering from cecal dilatation [9]. The absence of ping sound on auscultation with distension of cecum on per rectal examination was the confirmation for cecal impaction. Rectal palpation was a more reliable diagnostic tool than percussion auscultation in differentiating cecal impaction from cecal dilatation. Rectal palpation allowed an accurate diagnosis in all cases as also reported by Braun *et al*. [2]. The mean±SE values of hemoglobin, packed cell volume, and total leukocytic count of all animals with cecal affection were lying within their normal reference range for healthy animals which corroborated with the findings of Mohan *et al*. [10] who reported that packed cell volume and hemoglobin remained within normal limits in buffaloes during intestinal obstruction. In contrast to that, Papadopoulos *et al*. [11] reported that animals suffering from intestinal obstruction generally showed the higher value of hemoglobin and packed cell volume due to dehydration-related hemoconcentration. The marked neutrophilic leukocytosis observed in all the animals may be due to catecholamine or glucocorticoid response [12]. In the present study, hematological profile was found to be non-specific in the diagnosis of cecal dilatation and cecal impaction in bovine. Hypoproteinemia and hypoalbuminemia may be due to decreased dietary intake during gut diseases. Similar findings have been reported by earlier workers also [7,10]. Hypokalemia recorded in the present study may be due to be lack of ingestion of potassium associated with ↱anorexia [12]. Continuous secretion of chloride ions as hydrochloric acid from abomasal mucosa, reflux of ↱abomasal contents, inflow of saliva into the reticulorumen, and decreased absorption of chloride due to ↱obstruction, all contribute to accumulation of chloride ions in a compartment proximal to ↱obstruction or so-called third space which results in decreased chloride concentrations in plasma [13]. No appreciable variation was found in sodium which corroborated with the findings of Mohan *et al*. [10]. The phosphorous level was recorded within normal reference range for healthy animal, whereas calcium level was decreased and correlated with the findings of Braun *et al*. [5] and Hussain *et al*. [14]. However, Stocker *et al*. [15] were not certain whether the hypocalcemia was a cause or a result of cecal dilatation. They were of the opinion that intestinal obstruction might result in increased myoelectrical activity of the intestine, resulting in increased consumption of calcium, and hypocalcemia may result from cecal dilatation. Increased lactate in all the animals correlated with the findings of Hussain *et al*. [14]. Mild increase in serum lactate levels in the present study suggested that minimal mesenteric ischemia had occurred. This was also reflected by the good surgical outcome. Allen and Holm [16] reported that serum lactate concentration is the best marker of mesenteric ischemia. They opined that D-lactate is produced by intestinal bacteria after ischemia while L-lactate is released in increased amounts during hypoxia by anaerobic metabolism, so its measurement may be used for prognostic and therapeutic purposes. Increased total protein contents in the peritoneal fluid corroborate with the findings of Saini *et al*. [17]. Smith [12] reported that increased total protein contents in the peritoneal fluid might be associated with the leakage from the congested vessels of the obstructed segment, whereas low albumin could be due to low albumin level in the blood. Recently, ultrasonography had been used for the diagnosis of several gastrointestinal tract affections in buffalo [1]. Therefore, this study tried to use ultrasonography in the diagnosis of cecal affections in bovine. Ultrasonographic visualization of thin cecal wall is in agreement with earlier findings of Khalphallah *et al*. [1], Amrein [4], Braun *et al*. [5], Singh [7], and Imran and Tyagi [18]. The presence of reverberations across the cecal wall was indicative of gas in the cecum. Reverberation artifacts are associated with luminal gas in the visceral organs [19]. Visualization of cecal wall along with gas over a large area was confirmatory for cecal dilatation as reported by Khalphallah *et al*. [1], Singh [7], and Imran and Tyagi [18]. Ultrasonographic visualization of cecal wall along with strong distal acoustic shadow was confirmatory for cecal impaction. To the author’s knowledge, no reports on ultrasonographic features of cecal impaction are available in literature. In animals with cecal dilatation, left flank laparorumenotomy helped in retrieval of foreign bodies in two animals and reducing the volume of rumen for easy exploration of the right side of abdomen. This also helped in massaging the cecum and kneading the fecaliths present in the colon. Exteriorization of cecum was not required, and the technique was easy to perform and kneading could be completed within 2-3 min. Foreign bodies in the reticulum are incidental findings in bovine due to their ↱indiscriminate feeding habits and do not appear to be related to cecal dilatation as correlated with ↱the findings of Singh [7]. Cecal dilatation may also, secondarily, be associated ↱with foreign body syndrome [3]. The successful surgical outcome in five of six animals suggested that left flank exploration after laparorumenotomy followed by kneading of cecum for 2-3 min may be an ideal surgical technique for the management of cecal dilatation in bovine. Cecal dilatation and impaction conventionally required right flank laparotomy through mid-flank approach, exteriorization of cecum, and cecotomy as described in earlier workers [3,20]. However, conservative treatment of cecal dilatation using left flank approach of rumenotomy followed by kneading the cecum through the same incision has not been described in literature. Overall prognosis of cecal dilatation is fair with resumption of good health and normal production level of the survivors [3]. Right flank typhlotomy was found successful in management of cecal impaction in four of five animals. A high success rate (four of five animals) in management of cecal impaction cases by right flank typhlotomy suggested that, once cecal impaction is confirmed by per rectal examination and ultrasonography, right flank typhlotomy should be performed to decompress the cecum and to restore the patency of the large intestine.

## Conclusion

Cecal dilatation and cecal impaction can be diagnosed on the basis of clinical examination, percussion auscultation, and per rectal examination while ultrasonography can be used as a differential diagnostic tool. Percussion auscultation and per rectal examination are concluded as good diagnostic tools. Left flank exploration after laparorumenotomy is an ideal surgical technique for the management of cecal dilatation, while right flank typhlotomy is ideal for the management of cecal impaction in bovine.

## Authors’ Contributions

GS, as MVSc Scholar, designed this clinical study under the guidance of RKU and AA. GS, RKU, JM, TS, and AK conducted clinical and ultrasonographic examination and performed surgeries. PV and NUD provided help in drafting the manuscript and data analysis. All authors read and approved the final manuscript.
